# Supporting social justice through equity‐based actions for a sustainable future in animal genetics (at the 39th International Society for Animal Genetics Conference)

**DOI:** 10.1111/age.13506

**Published:** 2025-01-28

**Authors:** Sadye Paez, Ntanganedzeni Olivia Mapholi, Lucky Tendani Nesengani, Susan J. Lamont, Samuel E. Aggrey, Olivier Hanotte, Cynthia D. K. Bottema, Clare A. Gill

**Affiliations:** ^1^ Neurobiology of Language The Rockefeller University New York New York USA; ^2^ College of Agriculture and Environmental Sciences University of South Africa Science Johannesburg South Africa; ^3^ Department of Animal Science Iowa State University Ames Iowa USA; ^4^ Department of Poultry Science University of Georgia Athens Georgia USA; ^5^ International Livestock Research Institute (ILRI) Addis Ababa Ethiopia; ^6^ School of Life Sciences The University of Nottingham Nottingham UK; ^7^ School of Animal & Veterinary Sciences University of Adelaide Roseworthy South Australia Australia; ^8^ Department of Animal Science Texas A&M University College Station Texas USA

**Keywords:** agriculture, animal genetics, diversity, equity, inclusion, mentoring, networking, storytelling

## Abstract

The 39th International Society for Animal Genetics conference (ISAG) was held for the first time in Africa under the theme ‘Animal genetics for a sustainable future’ in 2023. The conference convened scientists, policy makers, industry professionals, and students from interdisciplinary fields to share and discuss the latest developments in the space of animal genetics. Since its inception as a society, ISAG has sought to provide a platform advocating for a just and equitable future in animal genetics. At the 39th ISAG conference, this commitment towards furthering inclusion in animal genetic science was progressed with two new offerings to attendees. The first session guided discussions on the political, ethical, legal, socioeconomic, and cultural dynamics that present barriers to participating in and benefitting from the genomic and genetic science fraternity. This session also included principles of social justice, specifically equity, diversity, and inclusion, towards enacting fairness in an unfair world, and focused on constraints related to sustainability in animal genetics. The second session used the important tradition of storytelling to transfer knowledge and wisdom from experienced scientists to upcoming researchers. Experienced scientists shared lived experiences on educational and career paths, challenges, and opportunities, providing networking and opportunities for further mentoring. Here, we report on these equity‐based actions and their relevance to address the urgent continent‐specific and global disparities in animal genetics to move towards a sustainable future.

## ISAG MEETS ON THE AFRICAN CONTINENT FOR THE FIRST TIME IN ITS NEARLY 70‐YEAR HISTORY

In July 2023, the College of Agriculture and Environmental Sciences at the University of South Africa (UNISA), in collaboration with other local universities and research institutions, hosted the 39th International Society of Animal Genetics (ISAG) Conference for the first time on the African continent. As a scientific society, ISAG provides a forum for exchange of research ideas, results, and applications between members. Nearly 600 international delegates, including but not limited to genetic and genomic scientists, policy makers, industry, government representatives, professionals, farmers, and students, gathered in Cape Town, South Africa, at plenary lectures, symposia and oral sessions, workshops, poster presentations, and networking across the theme, ‘Animal genetics for sustainable future.’

## SMALLHOLDER FARMS, CAPACITY DEVELOPMENT, AND KNOWLEDGE TRANSFER: KEYS TOWARDS SUSTAINABILITY IN AGRICULTURE

A sustainable future in animal genetics will require addressing the inequities and injustices in the broad scientific enterprise (Graves Jr et al., 2022; Haelewaters et al., 2021) and within agriculture (Food and Agriculture Organization of the United Nations, 2020; Sihlobo & Qobo, 2021). Agriculture is vital to global health and wellbeing and also serves as the backbone of most economies in African countries. Smallholder farms account for an estimated 80% of food production and provide livelihoods for over 2 billion people (Odiwuor, 2022; Food and Agriculture Organization of the United Nations, 2015), which are crucial for global food security and nutrition (Gomez et al., 2020). However, there are numerous systemic barriers to participation and sustainability, such as the cycle of low‐intensity, subsistence‐oriented farming that result in low yields (Gomez et al., 2020; Meemken & Bellemare, 2020), insufficient profits (Giller et al., 2021), land access (Żmija et al., 2020), property rights (Davies et al., 2021), and a host of other factors (Kim et al., 2021; Lee & Gambiza, 2022).

For example, in South Africa, communal farmers do not typically join local breeding programs because, historically, the design of these programs is most suitable for commercial farmers, typically white males, as a result of a long history of segregation policies and apartheid (Sihlobo & Qobo, 2021; Tyeni et al., 2020). Policies and institutional measures, such as the 1912 Land Bank Act (National Archives & Records Service of South Africa, 1912), 1913 Land Act (also named Natives Land Act) (South African Government, 2024), 1926 Agricultural Credit Act, and 1968 Marketing Act (J. University of the Witwatersrand, 2024) contributed to disparities between well‐resourced white‐owned commercial farms compared with poorly resourced small‐scale and subsistence black‐owned farms by providing financing, land access, and single channel marketing systems with guaranteed market access and prices for white farmers. Similar historical discrepancies of limited access to land, technology, and financing face women in African farming (Ugwu, 2019).

Today, this legacy continues and is a persisting barrier for communal farmers who want to enter commercial breeding programs or related breeding societies (Zantsi et al., 2019). Gendered agricultural tasks are also problematic, with women comprising 62% of the agricultural labor force while maintaining household and societal obligations (Kamau‐Rutenberg 2018) and being less likely to own the land (less than 20% globally) or livestock (Food and Agriculture Organization of the United Nations, 2020; Nagothu, 2019). Additionally, in other parts of the continent, females are discouraged from involvement in livestock farming due to continuing cultural beliefs that women cannot enter the kraals, traditional African villages or enclosures for livestock, because it will reduce animal performance (Beinart & Brown, 2013; Coertze, 1986; Gumede et al., 2018). Often viewed as the family's economic storehouse or its source of wealth, this challenge in accessing a kraal (also spelt craal) points to the patriarchal nature of these spaces that further marginalize women (Banda & Van der Merwe, 2017). A complete inclusion approach would, at a minimum, need to focus on communal and female farmers.

Capacity development and knowledge transfer are other important systemic barriers that must be addressed on the continent. There are few opportunities for smallholder and communal farmers to enter the market and participate in breeding programs; yet community‐based breeding programs realize sustainable genetic gains and economic benefits (Agricultural Research Council, 2019; Ambaw et al., 2023; Hunde et al., 2024; Voss et al., 2023). For instance, African‐based and African‐led projects aiming to build and develop capacity serve to enhance the computing capacity of African scientists, farmers, academics, and students. Some of these programs include the African BioGenome Project (AfricaBP) (Sharaf et al., 2023, 2024) and Human Heredity and Health in Africa (H3Africa) initiatives (Croxton et al., 2023). AfricaBP, a coordinated pan‐African effort, aims to build capacity to generate, analyze, and deploy genomics data for the improvement and sustainable use of biodiversity and agriculture across Africa. H3Africa focuses on empowering African scientists to be competitive in genomic sciences and to foster effective collaborations among African scientists through training and workshop programs for knowledge transfer. Despite these efforts, the overall agricultural structure in Africa is still largely dominated by males, severely underestimating the leadership of females and younger generations and their knowledge of indigenous breeding and specific sociocultural contexts.

These examples highlight the urgency in addressing inequities and injustices with smallholder farms, capacity development, knowledge transfer, and other barriers that threaten our global ability to meet the Sustainable Development Goals (Dang & Serajuddin, 2020; Moyer & Hedden, 2020) to end hunger, achieve food security and improved nutrition, and promote sustainable agriculture. Multiple agreements, in addition to the Sustainable Development Goals, support such transformative goals, including the Convention on Biological Diversity in its Kunming‐Montreal Global Biodiversity Framework, which outlines necessary steps towards ‘Living in harmony with nature by 2050’ (Convention on Biological Diversity, 2023). Target 10 supports sustainability in agriculture, and targets 20–23 underpin the necessity of promoting development of capacity building, establishing access to innovation and technical and scientific cooperation, and ensuring full, equitable, inclusive, effective, and gender‐responsive representation and participation in decision‐making by Indigenous Peoples and Local Communities.

## EQUITY‐BASED ACTIONS BEFORE AND DURING THE 39TH ISAG CONFERENCE

Intentional efforts addressing the continent‐specific and global disparities in animal genetics are urgent. Prof Puleng LenkaBula, Principal and Vice‐Chancellor of UNISA, outlined the importance of such deliberate measures to open the 39th ISAG conference: “While science is important, it cannot exist for its own sake; it must have a utilitarian value that responds to the challenges of society, its economic systems, social systems, and its interface with society and the future.” Diversity, equity, and inclusion (Rebbeck et al., 2022) are principles of justice at these societal interfaces that seek to promote opportunities and mitigate barriers to participating in and benefiting from genomic science; justice, in brief, is about creating fairness in an unfair world. Social injustices within the tradition of western science, which is embedded with structural racism and systemic oppression, historically excludes many communities (Amano et al., 2023; Cech, 2022; Godin, 2007; Harris & Nicolazzo, 2020; Mattison et al., 2022; Rippon, 2023; Taylor, 2020), contributes to ‘helicopter science’ practices (Haelewaters et al., 2021), and drives epistemic exclusion, including tokenism, marginalization, and racial microaggressions, broadly (Settles et al., 2021) and in the context of the African continent (Guedes et al., 2023).

Since its inception as an organization nearly 70 years ago, ISAG, albeit informally, has supported creating a just, equitable, and inclusive future in animal genetics. For instance, in an effort to support animal genetic research more globally, workshops on ‘Livestock genomics in developing countries’ have been held at ISAG conferences since 2012. At this 39th conference, the organizers augmented this commitment with actions before and during the meeting; here, we briefly detail these equity‐based efforts.

Before the conference took place, the ISAG leadership committed to further increasing the inclusion of the next generation of students. Their presence was visibly amplified at this conference, with over 100 bursaries awarded to students to attend the meeting, more than double in the past 10 years. Each bursary recipient was given an opportunity to share their science, either in an oral session or a poster presentation.

In conjunction with the local organizing committee, ISAG also accepted two new proposals for sessions: a ‘Justice, equity, diversity, and inclusion’ symposium and a ‘Words of Wisdom’ workshop. The first session, ‘*Decolonizing Science: A primer on centering justice, equity, diversity, and inclusion within animal genetics and genomics*’, invited delegates to consider the why and what of fulfilling the scientific potential of animal genetics (Cao et al., 2021; Chapman et al., 2022; Ghazal et al., 2021; Li et al., 2023) by addressing the web of cultural, political, ethical, legal, and socioeconomic issues contributing to the unsustainable development of biodiversity (Efferth et al., 2019; Haelewaters et al., 2021; Lythreatis et al., 2022; Smessaert et al., 2020; Sudoi et al., 2021) including agriculture (Biswas et al., 2023). This symposium asked participants to consider structural racism and systemic oppression in the broad scientific enterprise as well as individual and institutional power and positionality. Equity, diversity, and inclusion were outlined as a set of social justice principles with an urgency towards integration of these principles as core, technical components of science as a path forward for addressing injustices and inequalities.

The second session, ‘*Words of Wisdom: Engaging with Future Generations*’, included a moderated panel of four delegates sharing their lived experiences and journeys in science (Table 1). The goal of the workshop was to share accumulated wisdom from the perspective of ‘senior’ scientists, defined here as individuals who are further advanced in their careers with more experience or qualifications, specifically with extensive engagement with ISAG and the discipline of animal genetics. The format included the important cultural tradition of storytelling to transfer knowledge to future generations (Ruedas‐Gracia et al., 2022; Suzuki et al., 2018). By intentional design, the speakers collectively represented a diversity of scientific expertise, geography, gender, and race. The key learnings for future generations included sharing personal experiences regarding why and how animal genetics serves humankind and how diversity enhances the scientific enterprise. Additionally, panelists highlighted how pathways to successful careers can look very different for different individuals, such as the resiliency for persevering through personal challenges, importance of identifying and remaining true to one's core values, and significance of networking locally and globally for confidence, career advancement, and social well‐being.

**TABLE 1:** Summary from the ‘Words of Wisdom’ workshop sharing the different pathways and learnings from four ‘senior’ scientists.

	Educational path and current role	Challenges (financial, emotional and mental support, ‘failures’)	Examples of risks the individual took to grow/learn (tips!)	Opportunities offered from others that the individual engaged/responded to (who helped you and what did they offer… what did it require of you?)
S. J. Lamont	Education: BA at small liberal arts college; PhD at a public medical center Current role: Distinguished Professor	First generation college student First female faculty in department Sexual harassment from other faculty	Pursued jobs and roles in which I was a gender‐minority Joined sport team (for which I had NO talent) to network with colleagues	Undergrad professor included me on team to do field research, including travel to another country with otherwise all‐male team Post‐doc mentor gave me freedom to develop research topic and mentor grad students Colleagues helped me to network in my discipline
S. E. Aggrey	Education: BSc, MSc and PhD in three different public institutions in three different countries on three different continents Current role: Distinguished Professor/Russell Chair	First minority faculty in my department. There was a low expectation of me to succeed	Pursued agriculture when I had no clue about agriculture. I didn't know the difference between a broiler and a layer chicken	Undergrad/Grad School Great mentorship from my thesis/dissertation advisors Post‐doc mentor treated me like an independent scientist. Offered me a PhD student to supervise Faculty mentors from other institutions greatly helped me navigate the grant writing nuances and expand my network
O. Hanotte	Education: License (B.Sc.) en Science Zoologiques et Doctorat (PhD) en Science in Belgium; post‐doctoral fellowships in UK and Kenya. Current role: Professor of Genetic, UK, Principle Scientist, ILRI, Ethiopia	French native speaker in Anglophone countries European national in African countries	The world is yours, never be stopped by boundaries Learn the culture of the countries you are living in Learn the history of the countries you are living in	Open‐minded supervisors gave me the freedom to build up my scientific path. The best way for a scientist to flourish is to give them freedom of thought and initiative
C. Bottema	Education: BA and PhD at two USA state universities and two post‐doctoral fellowships at medical schools Current role: University adjunct professor in Australia	Discouraged from going to medical school because undergraduate grades were not exceptional so changed career direction	Not allowed to experiment on humans so switched from medical genetics to livestock genetics knowing very little about livestock This choice meant immigrating to Australia and having much less funding available, but absolutely worth the change because of job satisfaction knowing your science is making a real difference to farmers	Fantastic PhD supervisor who expected everyone in group to help each other with papers, experiments, talks, grants, etc. so we learned all aspects of being good scientists and mentors Post‐doc mentors, collaborators and colleagues were equally supportive but also challenged me to take on new research directions and academic roles

*Note*: Overlapping themes include the importance of networking locally and globally, the perseverance required to overcome challenges, the integrity of remaining true to one's core values, and the significance of diversity towards embracing the scientific enterprise and bettering outcomes in animal genetics.

The 39th ISAG conference included delegates (*n* = 571) from 50 countries, spanning every populated continent. An estimated 80 and 100 delegates attended the Decolonizing Science and Words of Wisdom workshops, respectively, with majority representation from early‐career scientists and participants from developing countries.

## ISAG'S COMMITMENT TOWARDS FORMALIZING A STANDING COMMITTEE ON EQUITY AND JUSTICE

Looking forward, the 40th ISAG Conference in Daejeon, South Korea in 2025, offers an opportunity to continue building on the continuum of progress from this most recent meeting. As a society, ISAG has a formal process for establishing standing committees (Article 21, ISAG Constitution, https://www.isag.us/docs/ISAG_Constitution.pdf?v2). So that these equity‐based efforts can continue in the future, a request has been submitted to host these workshops in Daejeon and is being reviewed by the 2025 Local Organizing Committee. Additionally, a request will be submitted to the ISAG Board in Daejeon to recognize an ‘Equity and Justice’ standing committee, which would be voted on by the membership at the general business meeting concluding the conference. Other opportunities for further inclusion and participation are being explored, such as adding live closed captioning during each plenary session and coordinating parental assistance (e.g., lactation rooms, childcare support). These actions align with the ISAG Members Survey Summary of Responses (https://urldefense.com/v3/__https://www.isag.us/business.asp__;!!M59pwtRysTUf!ZzgakXXYz8mZS3eBccqfNuRg5hgyiNuf_JifyR2se‐gnYfvm3Zxfd6dTgOZ220ZSXBVcwUL7TCol6MWmhhAV5X11_K9jCfLU$) that reported a desire for increased professional development for early career scientists with increased financial, career development, and communications support (e.g., training workshops and accompanying courses such as Massive Open Online Courses on techniques and experimental designs, travel awards and ‘real’ awards for young investigators, course development, and mentoring/collaborations talks, increased on‐line and social media use to engage diverse, tech‐savvy audiences).

Promoting a transparent and intentional approach to increase access, build capacity, and elevate a diversity of voices and perspectives for all people involved in animal genetics is critical for responding to the challenges of society. Here, we have shared the actions by ISAG at the 39th conference and intentions for the next conference, demonstrating the role of ISAG in leading a sustainable future in animal genetics.

## AUTHOR CONTRIBUTIONS

**Sadye Paez:** Conceptualization; writing – original draft; writing – review and editing; visualization; supervision. **Ntanganedzeni Olivia Mapholi:** Conceptualization; funding acquisition; writing – original draft; writing – review and editing. **Lucky Tendani Nesengani:** Writing – original draft; writing – review and editing. **Susan J. Lamont:** Writing – original draft; writing – review and editing. **Samuel E. Aggrey:** Writing – original draft; writing – review and editing. **Olivier Hanotte:** Writing – original draft; writing – review and editing. **Cynthia D. K. Bottema:** Writing – original draft; writing – review and editing; visualization. **Clare A. Gill:** Funding acquisition; writing – review and editing.

## CONFLICT OF INTEREST STATEMENT

C.A.G. served as the President of ISAG and Chair of IFAG from 2019 to 2023.

## Data Availability

This paper provides data related to conference attendance based on registration. The data in the table was completed by its respective authors.
